# Is the Evolution of *Salmonella enterica* subsp. *enterica* Linked to Restriction-Modification Systems?

**DOI:** 10.1128/mSystems.00009-16

**Published:** 2016-06-21

**Authors:** Louise Roer, Rene S. Hendriksen, Pimlapas Leekitcharoenphon, Oksana Lukjancenko, Rolf Sommer Kaas, Henrik Hasman, Frank M. Aarestrup

**Affiliations:** a Division for Epidemiology and Microbial Genomics, National Food Institute, Technical University of Denmark, Kongens Lyngby, Denmark; b Section for Microbiology and Diagnostics, Statens Serum Institut, Copenhagen, Denmark; UC Davis Genome Center

**Keywords:** restriction-modification systems, evolution, *Salmonella* phylogenetic analysis, next-generation sequencing, whole-genome sequencing

## Abstract

*Salmonella enterica* subsp. *enterica* bacteria are highly diverse foodborne pathogens that are subdivided into more than 1,500 serovars. The diversity is believed to result from mutational evolution, as well as intra- and interspecies recombination that potentially could be influenced by restriction-modification (RM) systems. The aim of this study was to investigate whether RM systems were linked to the evolution of *Salmonella enterica* subsp. *enterica*. The study included 221 *Salmonella enterica* genomes, of which 68 were *de novo* sequenced and 153 were public available genomes from ENA. The data set covered 97 different serovars of *Salmonella enterica* subsp. *enterica* and an additional five genomes from four other *Salmonella* subspecies as an outgroup for constructing the phylogenetic trees. The phylogenetic trees were constructed based on multiple alignment of core genes, as well as the presence or absence of pangenes. The topology of the trees was compared to the presence of RM systems, antimicrobial resistance (AMR) genes, *Salmonella* pathogenicity islands (SPIs), and plasmid replicons. We did not observe any correlation between evolution and the RM systems in *S. enterica* subsp. *enterica*. However, sublineage correlations and serovar-specific patterns were observed. Additionally, we conclude that plasmid replicons, SPIs, and AMR were all better correlated to serovars than to RM systems. This study suggests a limited influence of RM systems on the evolution of *Salmonella enterica* subsp. *enterica*, which could be due to the conjugational mode of horizontal gene transfer in *Salmonella*. Thus, we conclude that other factors must be involved in shaping the evolution of bacteria.

**IMPORTANCE** The evolution of bacterial pathogens, their plasticity and ability to rapidly change and adapt to new surroundings are crucial for understanding the epidemiology and public health. With the application of genomics, it became clear that horizontal gene transfer played a key role in evolution. To understand the evolution and diversification of pathogens, we need to understand the processes that drive the horizontal gene transfer. Restriction-modification systems are thought to cause rearrangements within the chromosome, as well as act as a barrier to horizontal gene transfer. However, here we show that the correlation between restriction-modification systems and evolution in other bacterial species does not apply to *Salmonella enterica* subsp. *enterica*. In summary, from this work, we conclude that other mechanisms might be involved in controlling and shaping the evolution of *Salmonella enterica* subsp. *enterica.*

## Abstract (precis)

The evolution of bacterial pathogens, their plasticity and ability to rapidly change and adapt to new surroundings are crucial for understanding the epidemiology and public health. With the application of genomics, it became clear that horizontal gene transfer played a key role in evolution. To understand the evolution and diversification of pathogens, we need to understand the processes that drive the horizontal gene transfer. Restriction-modification systems are thought to cause rearrangements within the chromosome, as well as act as a barrier to horizontal gene transfer. However, here we show that the correlation between restriction-modification systems and evolution in other bacterial species does not apply to *Salmonella enterica* subsp. *enterica*. In summary, from this work, we conclude that other mechanisms might be involved in controlling and shaping the evolution of *Salmonella enterica* subsp. *enterica.*

## INTRODUCTION

The *Salmonella* genus is highly diverse even though it is comprised of only two species, *Salmonella enterica* and *Salmonella bongori*. The species *S. enterica* contains six subspecies, of which the highly diverse members of *S. enterica* subsp. *enterica* can be subdivided into more than 1,500 serovars. This subspecies is a common cause of diseases in humans and domestic animals (1, 2) and one of the leading causes of foodborne illness worldwide (3).

Recombination between genomes is thought to be a major driver in evolution (4) and to contribute to the diversity within the *Salmonella* genus (5, 6). It was suggested that in *Neisseria meningitides*, the phylogeny is associated with the content of restriction-modification systems (RM systems) (7). Furthermore, rearrangements of genomes caused by RM systems are described as factors that could influence the evolution of pathogens (4). In addition to their role in rearrangements, RM systems are also considered to be a barrier for horizontal gene transfer (HGT) between bacteria, thus serving as an immune defense system for uptake of foreign DNA (8–10). The contribution, to our knowledge, has never been quantified, and thus, we have recently shown that for conjugational transfer between isogenic *Escherichia coli* isolates, the barriers of RM systems are not absolute (11).

RM systems are comprised of a restriction enzyme (RE) and a cognate methyltransferase. The restriction enzyme recognizes and digests foreign incoming DNA, whereas the methyltransferase performs methylation of the bacterium’s own DNA to protect itself from degradation by the cognate restriction enzyme (12, 13). This enables the bacterium to distinguish between its own (methylated) DNA and incoming nonmethylated DNA.

The RM systems are divided into four types (I to IV), based on their protein complexes, the subunit composition, and the functionality of the system (14). The type I systems are complexes of three gene products: *hsdR* (R, restriction), *hsdM* (M, methylation), and *hsdS* (S, sequence specificity). This type cleaves nonmethylated DNA randomly at a remote distance from the recognition sequence determined by the specificity subunit. The protein complex of all three gene products, R_2_M_2_S (two subunits of R and M and one subunit of S) must be established prior to restriction, whereas a complex of only the *hsdM* and *hsdS* proteins (M_2_S) is needed for methylation of the DNA (15). Type II systems are only made up of methyltransferases and restriction enzymes, where the function and composition of the M and R products varies depending on the subtype of the system. Type II systems modulate (cleave and/or methylate) unmethylated DNA at specific recognition sites, making them suitable as molecular biological tools to cut DNA for cloning or other analysis where only a piece of DNA is needed (12, 15). Type III systems, consisting of the gene products Res and Mod, hemimethylate the DNA and cleave DNA at specific sites 25 to 27 bp downstream from the recognition sequence (16), whereas type IV, compared to types I to III, does not encode a methyltransferase and only cleaves methylated DNA (12, 15).

In this study, we elucidated the potential association between RM systems and the phylogeny of *S. enterica* subsp. *enterica* serovars. We tested the hypothesis that RM systems might be linked to the evolution of *S. enterica* subsp. *enterica* and thereby be responsible for the diversification of the species. The most effective source of variation within the genome is caused by HGT (17), transferring, e.g., antimicrobial resistance (AMR) genes between bacteria. In *Salmonella*, the *Salmonella* pathogenicity islands (SPIs) are believed to be acquired by horizontal gene transfer and to have an effect on the structure of the genome (18, 19). Thus, we also elucidated the content of plasmids, AMR genes, and SPIs in correlation to the RM systems and the phylogeny of the species to examine their effect on the evolution.

## RESULTS

### Genomes.

A total of 221 *Salmonella* genomes were included in the analysis. One hundred fifty-three genomes previously described by Timme et al. (1) were retrieved from the European Nucleotide Archive; their accession numbers are listed in Table S1 in the supplemental material. This collection was merged with 68 genomes sequenced as part of the 100K Foodborne Pathogen Genome Project (http://100kgenome.vetmed.ucdavis.edu/, NCBI BioProject accession number PRJNA186441; individual accession numbers are listed in Table S1). The final collection consisted of 216 *S. enterica* subsp. *enterica* genomes and five genomes of other *S. enterica* subspecies. The 221 *Salmonella* genomes are summarized in the supplemental material (see Table S1).

10.1128/mSystems.00009-16.5 Table S1  Full genomic information. Download Table S1, XLSX file, 0.1 MB. Copyright © 2016 Roer et al. 2016 Roer et al. This content is distributed under the terms of the Creative Commons Attribution 4.0 International license.

### Characterization of restriction-modification systems.

To characterize the RM systems of the 221 genomes, a whole-genome-sequencing (WGS) analysis was performed using the newly developed tool Restriction-ModificationFinder 1.0. Partial systems were completed by individual BLAST analysis of up- and downstream sequences against the REBASE database (20). In total, we identified 113 putative RM systems, including 58 type I RM systems (TI), of which 43 had unknown recognition sequences, 23 type II RM systems (TII), 2 type III RM systems (TIII), and 30 type IV RM systems (TIV). In addition, numerous methyltransferases outside the RM systems were identified, including type I, type II, and type III methyltransferases (see Table S2 in the supplemental material).

10.1128/mSystems.00009-16.6 Table S2  Overview of genes in the RM systems. Download Table S2, XLSX file, 0.1 MB. Copyright © 2016 Roer et al. 2016 Roer et al. This content is distributed under the terms of the Creative Commons Attribution 4.0 International license.

The additional methyltransferases (without associated restriction enzymes) identified were not included in the analysis, as the barrier for HGT is thought to be caused by the cleavage from REs.

**FIG 1:**
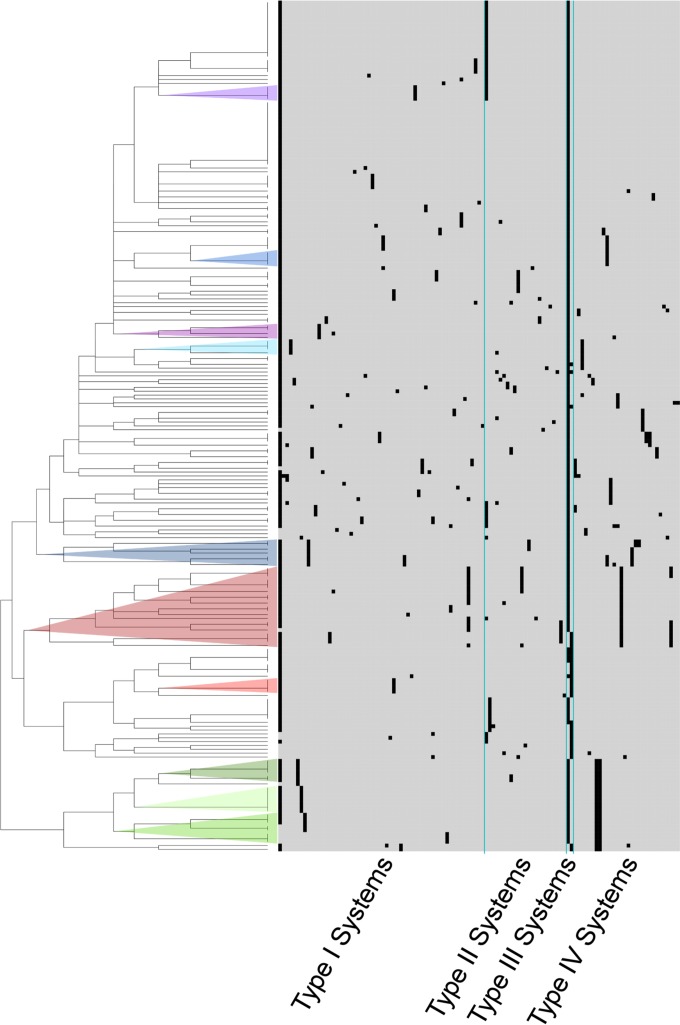
Presence or absence matrix of the 113 restriction-modification systems. In the matrix, each row represents one genome analyzed, and each column represents one of 113 RM systems. The cladogram is a hierarchical clustering of the genomes based on the Euclidean algorithm. Convergence of genomes with highly similar RM system contents and discrete phylogenetic clades on the core and pangenome trees is indicated by the colors in the cladogram.

One of the genomes only contained one RM system, while the other genomes contained between two and seven systems. All genomes contained a type III RM system, one of which was shared by 198 genomes. The type I system TI-1 was shared by 203 of the genomes, and 37 RM systems were specific to a single genome. The remaining systems were shared by 2 to 38 genomes. The distribution of the RM systems is illustrated in the presence/absence matrix in Fig. 1 and presented in detail in Fig. S1. The analysis revealed very diverse content of RM systems, and in assessing the highest level of discrimination in the cladogram, 120 distinct clusters were formed, with 77 clusters containing a single genome.

10.1128/mSystems.00009-16.1 Figure S1  Distribution of RM systems in *Salmonella enterica* isolates. Download Figure S1, TIF file, 2.6 MB. Copyright © 2016 Roer et al. 2016 Roer et al. This content is distributed under the terms of the Creative Commons Attribution 4.0 International license.

### The salmonella pan- and core genomes.

The pan- and core genomes were estimated based on the 221 *S. enterica* genomes. The progression of the pan- and core genomes as increasing numbers of the *S. enterica* genomes were added to the analysis is shown in Fig. S2 in the supplemental material. In analyzing the pangenome, consisting of any gene families found, the gene families increased gradually with the one-by-one addition of the different *S. enterica* subsp. *enterica* serovars, compared to a distinct increase in the number of gene families with the inclusion of *S. enterica* subsp. *diarizonae*. In contrast, the number of conserved gene families across the *S. enterica* subsp. *enterica* genomes in the core genome analysis was relatively consistent; however, the number of core families dropped when other *S. enterica* subspecies were introduced into the analysis. The final analysis comprising all 221 *S. enterica* genomes contained 16,375 gene families in the pangenome (for a representative genome, see Text S1 in the supplemental material) and 2,138 gene families in the core genome (for a representative genome, see Text S2). Analyzing the total number of gene families in the pangenome, each *S. enterica* genome contributed, on average, 65 new gene families, increasing the diversity within the *S. enterica* species.

10.1128/mSystems.00009-16.2 Figure S2  Pan- and core genome plot of 221 *Salmonella enterica* genomes. Download Figure S2, TIF file, 1.8 MB. Copyright © 2016 Roer et al. 2016 Roer et al. This content is distributed under the terms of the Creative Commons Attribution 4.0 International license.

10.1128/mSystems.00009-16.7 Text S1  Representative genome of the 16,375 *Salmonella* pan genes in FASTA format. Download Text S1, TXT file, 6.3 MB. Copyright © 2016 Roer et al. 2016 Roer et al. This content is distributed under the terms of the Creative Commons Attribution 4.0 International license.

10.1128/mSystems.00009-16.8 Text S2  Representative genome of the 2,138 *Salmonella* core genes in FASTA format. Download Text S2, TXT file, 1.1 MB. Copyright © 2016 Roer et al. 2016 Roer et al. This content is distributed under the terms of the Creative Commons Attribution 4.0 International license.

### The link between evolution and restriction-modification systems.

**FIG 2:**
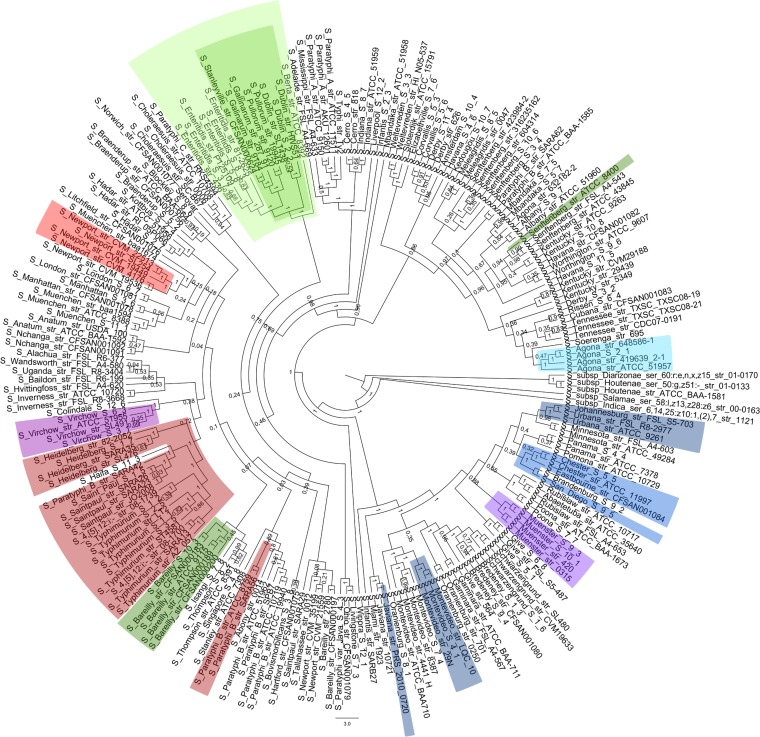
Concatenated core genome tree of *Salmonella enterica* subspecies *enterica* serovars constructed on 1,072 core gene clusters. The phylogenetic tree is constructed on core genes and represented as a cladogram. Discrete phylogenetic clades with highly identical RM system contents are indicated by the colors defined in Fig. 1.

**FIG 3:**
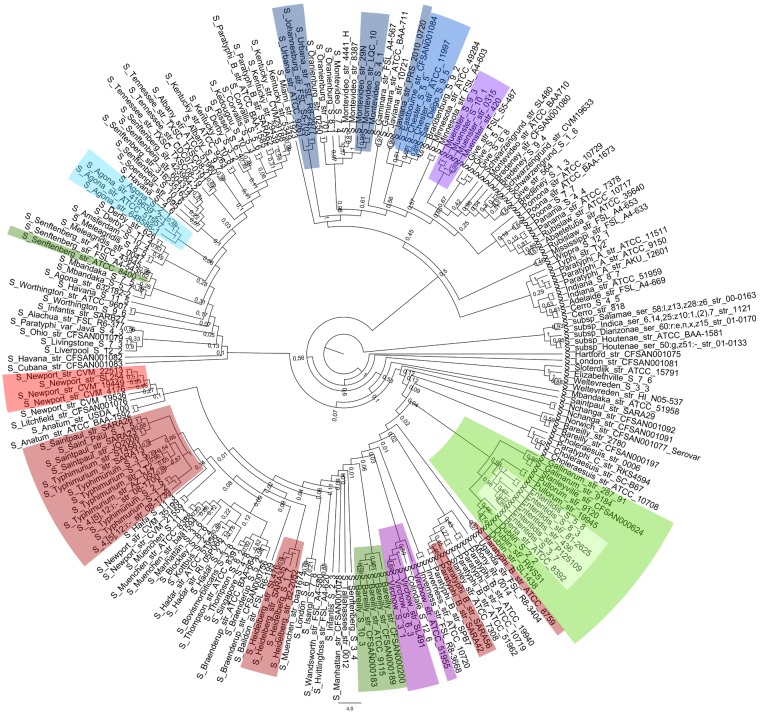
Pangenome tree of *Salmonella enterica* subspecies *enterica* serovars with RM systems indicated by colors. The phylogenetic tree is constructed from the presence/absence matrix of genes across genomes and represented as a cladogram. The colors represent the different groups of RM systems defined in Fig. 1.

To study the genomic evolution of *S. enterica* subsp. *enterica*, differences within the core genes were examined for all 221 genomes, and the results are illustrated by the phylogenetic core genome tree in Fig. 2. This evolution of *S. enterica* subsp. *enterica* is formed not only by the differences in the genes shared by the genomes but also by the loss of genes leading to differences in gene content. Figure 3 displays the pangenome tree, based on the presence and absence of genes across all the genomes included in the study.

For both the core and pangenome trees, the high bootstrap values of 1 near the root of the trees reflected a good phylogenetic representation of the data of the *Salmonella* genus, whereas the low bootstrap values found in some of the branches indicated difficulties determining the definite arrangement within the branch. These difficulties could be caused by different possibilities, such as the genomes being highly similar in their core or pangene content, high levels of gene transfer or recombination, or convergent evolution. However, both the core and pangenome trees separated the serovars from each other.

To examine the hypothesis of RM systems being linked to the evolution of *S. enterica* subsp. *enterica*, parallel analyses were performed to identify groups of genomes with highly similar RM patterns that also formed discrete phylogenetic clusters on the core and pangenome trees. Genomes forming distinct clusters by their RM systems are indicated by different colors in the matrix of RM systems (Fig. 1) and in the core and pangenome trees (Fig. 2 and 3). A few small clusters with almost identical RM content, partly clustering together in the core and pangenome trees, were observed. In most cases, the genomes within a cluster of the same color belonged to the same serovar, as for *S. enterica* subsp. *enterica* serovar Bareilly or Agona (dark green and light blue). However, three larger clades, with 21, 8, and 7 genomes, were identified in the RM matrix. Of the 21 genomes in the first clade, containing *S. enterica* subsp. *enterica* serovars Typhimurium, Saint Paul, Paratyphi B, and Heidelberg, 90% of the genomes were located together in one distinct cluster in the core genome tree, and the remaining 10% were also located together. For the pangenome tree, the 21 genomes were identified at 4 different locations, with a distribution of 67%, 19%, 9%, and 5% of the genomes, respectively. The two larger clades of 8 and 7 genomes were located adjacent to each other in the RM matrix, the first containing *S. enterica* subsp. *enterica* serovars Stanleyville, Gallinarum, Pullorum, and Dublin and the second containing serovars Enteritidis and Berta. When comparing the two RM clades to the core and pangenome trees, the two clades clustered together with all 100% of the genomes in both trees, indicating evolutionary relatedness.

As all the genomes contained a type III system, it was hypothesized to be the first introduced into *S. enterica* subsp. *enterica*. The core and pangenome trees were compared with the phylogenetic relationship between the type III systems (see Fig. S4 in the supplemental material). From the visual inspection and comparison of the trees, we found a common distinct clade in the phylogeny of the type III systems (marked in Fig. S4) where 48 of the 49 genomes were located together in the core genome tree, whereas on the pangenome tree, 47 of the 49 genomes were located together in a distinct clade. No other correlations were observed between the type III systems and the phylogeny of *S. enterica* subsp. *enterica*.

### Plasmid replicons, antimicrobial resistance, and pathogenicity islands in *Salmonella enterica.*

All 221 *Salmonella* genomes were analyzed for their content of plasmid replicons by using the Center for Genomic Epidemiology (CGE) Web tool PlasmidFinder 1.2, a tool proven capable of detecting 100% of the previously characterized and fully sequenced plasmids applied to the analysis (24 plasmids), in addition to detecting a broad variety of plasmid replicons among a collection of *S. enterica* subsp. *enterica* serovar Typhimurium isolates (21). Out of the 221 genomes tested in this study, 118 did not contain any replicons present in the PlasmidFinder database. In the remaining 103 genomes, 40 different plasmid replicons were identified, with each genome containing up to seven different replicons.

**FIG 4:**
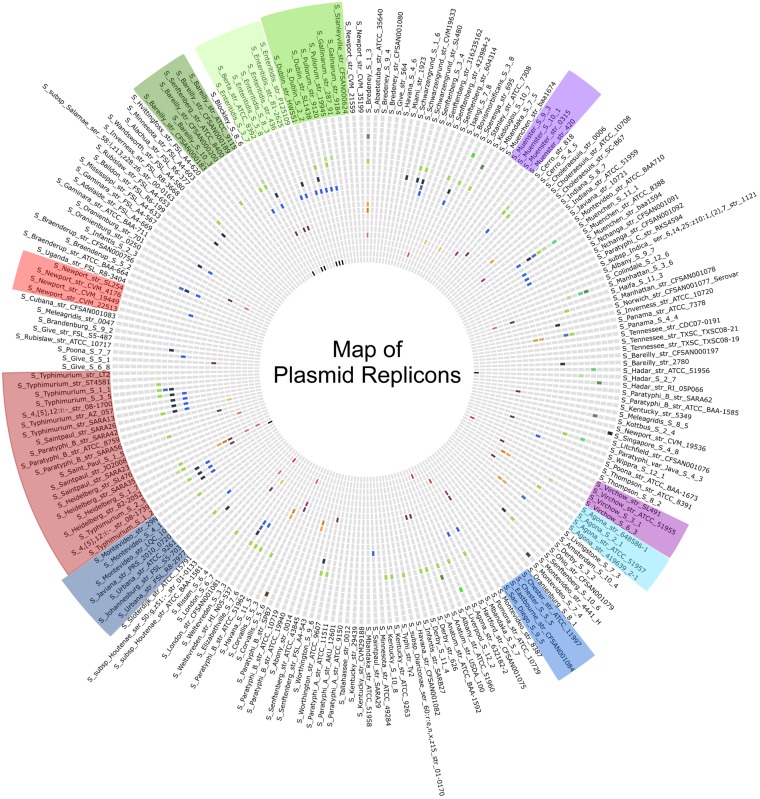
Map of plasmid replicons in *Salmonella enterica*. The genomes are ordered according to their RM systems and colored as in Fig. 1. The circles in the map represent the replicons, from the outside to the middle, *incA/C*, *incA/C2*, ColMGD2, Col156, Col8282, ColE10, ColpVC, ColRNAI, *incFIA*, *incFIB*, *incFIC*, *incFII*, *incHI1A*, *incHI1B*, *incHI2*, *incHI2A*, *incI1*, *incI2*, *incN*, *incP*, p0111, *incQ1*, *incR*, *incX1*, and *incX4*. In the plasmid replicon map, light gray indicates the absence of a replicon in the given genome, and the colors indicate the presence of a specific replicon.

Assessing the replicons in comparison with the RM systems observed in the *S. enterica* subsp. *enterica* serovars, no visual similarity was observed (Fig. 4), and RM systems and plasmid replicons were never observed on the same contigs. Evaluating the plasmid replicons for correlation to serovars, no correlation was observed between the serovars and the quantity of replicons; the 11 genomes with the highest replicon content represented 10 different serovars. However, for multiple isolates with the same serovar, common replicons were observed, such as with the *incFII* and *incX1* replicons present in all of the *S. enterica* subsp. *enterica* serovar Dublin genomes. Additionally, *incFII* was observed in 50% of the *S. enterica* subsp. *enterica* serovar Enteritidis genomes and, together with the *incFIB* replicon, in 50% of the *S. enterica* subsp. *enterica* serovar Typhimurium and both 4,[5],12:i: genomes. Interestingly, even though the numbers of replicons are not equal in identical serovars, the visual inspections imply an association between replicons and serovars and no association between replicons and RM systems.

AMR genes were found in 220 of the genomes, varying from 1 to 19 different genes per genome, with *aac(6′)-Iy* present as the only resistance gene in 140 of the genomes. The correlation of AMR genes and RM systems was examined (see Fig. S3 in the supplemental material); however, no visual conjunction was observed. Despite this potential bias, the assessment of correlation between AMR genes and plasmid replicons revealed different resistance genes located on the same contigs as plasmid replicons. Few genomes contained more than four plasmid replicons, and most contained between 10 and 16 resistance genes, correlating high resistance to the number of replicons present in the genomes.

10.1128/mSystems.00009-16.3 Figure S3  Distribution of antimicrobial resistance genes in *Salmonella enterica* isolates. Download Figure S3, TIF file, 2.6 MB. Copyright © 2016 Roer et al. 2016 Roer et al. This content is distributed under the terms of the Creative Commons Attribution 4.0 International license.

10.1128/mSystems.00009-16.4 Figure S4  Phylogenetic relationship of RM type III systems found in all genomes. Download Figure S4, TIF file, 2.5 MB. Copyright © 2016 Roer et al. 2016 Roer et al. This content is distributed under the terms of the Creative Commons Attribution 4.0 International license.

**FIG 5:**
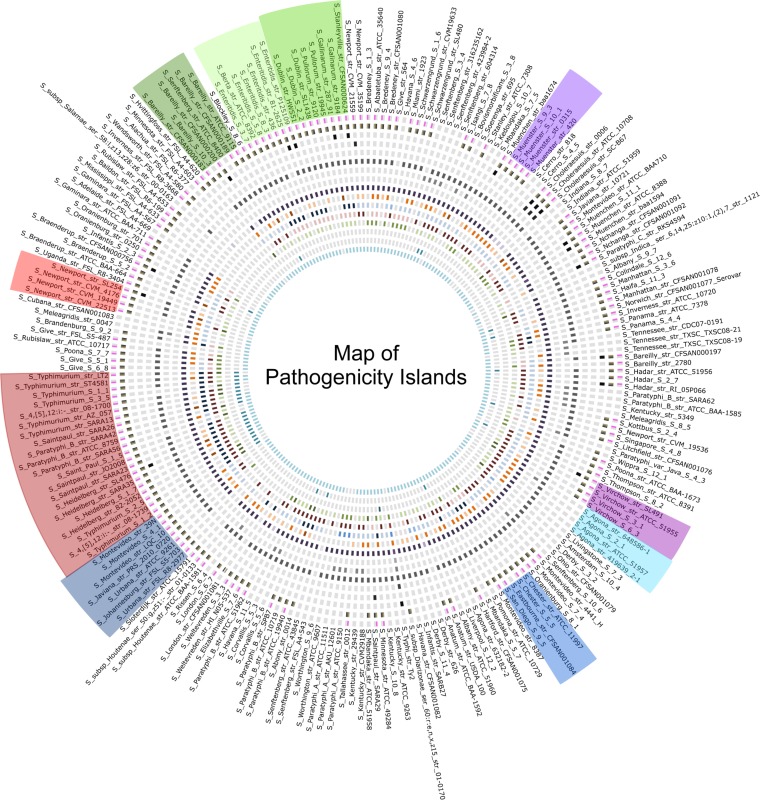
Map of *Salmonella* pathogenicity islands found in *Salmonella enterica* genomes. The genomes are ordered by their RM system profiles, indicated by the colors from Fig. 1. Each SPI is defined by a circle and colored according to the variant of the SPI. From the inner circle outward, the order of the SPIs is C63PI, CS54 island, SGI1, SPI-1, SPI-2, SPI-3, SPI-4, SPI-5, SPI-7, SPI-8, SPI-9, SPI-10, SPI-11, SPI-12, genes of SPI-13, and genes of SPI-14.

The presence of SPIs was assessed in all 221 *Salmonella* genomes by utilizing the newly developed Web tool SPI-Finder 1.0, and the results are visualized in Fig. 5. SPIs were found in all 221 genomes, and the number of SPIs in each genome varied from 1 to 14 islands/genes of islands.

The comparison of the SPIs and the content of RM systems revealed no clear association (Fig. 5). However, in associating the SPIs to individual *Salmonella* serovars, such as *S. enterica* subsp. *enterica* serovars Typhimurium, Paratyphi A, Choleraesuis, Heidelberg, and Saintpaul, association was apparent within the specific serovars.

## DISCUSSION

For decades, RM systems have been recognized for their ability to act as “immune systems” for bacteria, helping to determine whether or not foreign DNA was established in the cell. For *N. meningitidis*, the impact of RM systems in evolution was recently elucidated, and the purpose of the current study was to clarify whether a similar association could be identified in *S. enterica* subsp. *enterica*, by investigating a large subset of different whole-genome-sequenced *S. enterica* subsp. *enterica* serovars and an outgroup of five genomes from other subspecies. However, it was not possible to detect any significant association between RM systems and the overall evolution of *Salmonella enterica* subsp. *enterica*, even though we did observe genomes from the same RM clades in discrete phylogenetic clusters of both the core and pangenome trees.

The *Neisseria* genus, including *N. meningitidis*, is known to serve as a paradigm for natural transformation, where genetic exchange happens frequently due to their persistent competence independent of the phase of their life cycle (22). As RM systems have been recognized as barriers for transformation in multiple species (23–25), the link between a naturally transformable species and the RM systems, as shown for *N. meningitidis*, seems reasonable. However, transformation in *Salmonella* is unlikely, as they are not naturally transformable, and the transfer of genetic material happens mainly through conjugation (26). In addition, a recent study performed in *E. coli* (11) indicated that the barriers imposed by the RM systems in conjugational plasmid transfer were not absolute, which could explain the lack of significant association in our study when trying to associate RM systems to the plasmid replicons, AMR genes, SPIs, and their influence on the evolution.

The core and pangenome trees were constructed with a method previously described by Leekitcharoenphon et al. (27), where 73 genomes were evaluated. They found a core genome of 2,882 genes and a cognate pangenome of 10,581 genes. In our study, the core genome was found to comprise 2,138 genes and the cognate pangenome to contain 16,375 genes of the 221 genomes assessed. Additionally, judging from the pan- and core genome plot (see Fig. S2 in the supplemental material), the pangenome does not seem to be saturated. Hence, the pangenome is very likely to increase if additional genomes are added to the analysis, which indicates an open pangenome for *S. enterica* subsp. *enterica*, compared to the very uniform species *Bacillus anthracis*, having a closed genome, where the addition of genomes to the analysis will not increase the gene pool (28). This also supports the knowledge of *S. enterica* subsp. *enterica* being a highly diverse bacterial species.

Vasu and Nagaraja (4) recently described how changes in the specificity or the acquisition of new RM systems could alter the strains genetically from the original clonal population, as the methyltransferase modifies the genome of its new host and the RE prevents genetic exchange between closely related strains. Thus, mutations accumulate in the “new” strain, leading to genetic diversity. Assessing the RM systems in the 221 genomes, we identified 113 RM systems and numerous individual methyltransferases, with each genome harboring 1 to 7 RM systems. We found type III systems in all of the genomes, with 198 of the 221 genomes analyzed sharing a recognition sequence; the recognition sequences for the remaining isolates are yet unknown. Taking this result into consideration, it is plausible that the type III system was the first RM system introduced into *S. enterica* subsp. *enterica*, with subsequently a greater diversity following the later acquisition of new RM systems as described by Vasu and Nagaraja (4). However, the same RM systems could also have been introduced into different branches at different time points, resulting in identical RM system clades across the trees. To assess this hypothesis, a phylogenetic relationship of the type III systems was constructed and compared with the evolution depicted in the core and pangenome trees. We observed that a distinct clade on the type III system tree also recurred as distinct clades on both the core and pangenome trees, with high bootstrap values.

As the function probably is the same for all the type III systems found here (including the systems without recognition sequences assigned as yet), and as a type III system is found in all the genomes, we do not believe that the type III systems has contributed to the evolution, but the results could suggest that the type III system was the first introduced.

When further assessing the pan- and core genome trees according to all the RM systems present, there are indications of some clustering of genomes with similar RM systems, i.e., the cluster of *S. enterica* subsp. *enterica* serovars Enteritidis and Dublin, as well as the red RM system clade consisting of serovars Typhimurium and Heidelberg located together in both trees. However, the influence is not significant, indicating that the evolution could be driven by several factors. For instance, a previous study compared 28 *Salmonella enterica* isolates and provided evidence that clustered regularly interspaced short palindromic repeats (CRISPR) mediated sublineage evolution (29). Other drivers in evolution are host and environmental adaptations, which besides gene acquisition can be caused by gene loss and deletion, gene duplication, and changes within genes by, e.g., mutations (17, 30).

In the study on *N. meningitidis*, the researchers investigated the association of RM systems, homologous recombination, and the phylogenetic network (7). The main study was performed on 20 genomes, covering 5 serogroups out of the recorded 13 serogroups for *N. meningitidis* (31, 32). Budroni et al. found that genomes from the same clonal complex (CC) were located together in phylogenetic clades based on their core genes. In addition, the clades could be associated with the RM systems identified (7). In our study, we investigated 217 genomes of *S. enterica* subsp. *enterica*, including 97 different serovars, laong with an outgroup of five genomes from four other subspecies. Considering the highly diverse data set investigated in this study compared to the one used in the study of *N. meningitidis*, we observed small trends of sublineage association of RM systems and evolution. This could indicate that even though the data set investigated in this study was comprehensive, more genomes of each serovar should be included to cover the complete picture of the influence of RM systems in the evolution of *S. enterica* subsp. *enterica*. With the current speed in WGS, this might be realistic in the nearest future. Thus, even in the ideal data scenario, the lack of association is very likely due to the incomplete barrier of RM systems in conjugation.

As for all database-dependent approaches, the methods are only capable of detecting and reporting records present in the database explored. Our analysis for detecting the RM systems was limited to the current knowledge presented in the REBASE (33), where the recognition sequences of various type I-specificity subunits were not yet determined. Thus, it is likely that some strains have acquired RM systems with identical recognition patterns; however, this is presently unknown. This is why all of the database-dependent analyses should be interpreted with care.

The plasmid replicons which potentially could have an effect on the bacterial diversity due to horizontal gene transfer were also identified, but no clear correlation between the RM systems and the content of harbored plasmid replicons was observed. However, this approach might be complicated by the fact that plasmids are transferable and affected by factors such as fitness costs, selective pressure (34–37), and again, the fact that the RM barrier is not absolute (11). Thus, the analysis performed on the plasmid replicons illustrates the current status at the time of isolation, in contrast to what plasmids potentially could be acquired. AMR can be encoded by genes located on transferable plasmids; this potentially could reflect the promiscuity of the genomes reflected in current time, which could explain the lack of association between AMR genes and RM systems. This might also be explained by the possibility that the data set is biased with respect to selective criteria, e.g., susceptibility to antimicrobial agents, plasmid content, or virulence (SPI) of the isolates. However, the content of the RM systems is not believed to be affected by the possible biases.

The mechanism behind the acquisition of SPIs is horizontal gene transfer (18, 19). Nevertheless, the maintenance of SPIs within the genomes is considered stable (38) and is therefore a good measure of the barriers of RM systems compared to plasmid replicons and AMR genes, which can easily be lost if they do not confer any beneficial traits to the host. Despite this speculation, an influence of RM systems on the distribution of the SPIs was not supported by our analysis—on the contrary, there were indications of some SPIs being serovar specific, which corresponds to previous findings (38).

In conclusion, recombination and rearrangement events caused by RM systems are, in several cases, described as driving factors for evolution, contributing to the diversity within a species (4, 39–44). However, high recombination between two distantly related lineages of *S. enterica* is exceptional (6, 45), thus explaining the difficulties of linking the RM systems to the evolution of *S. enterica* subsp. *enterica*. Thus, recombination occurs within and between closely related serovars (6).

In this study, we showed that RM systems could not be linked to the evolution of *S. enterica* subsp. *enterica*, very likely due to the incomplete barriers of RM systems in conjugation. However, we observed closely related serovars with identical RM systems, i.e., *S. enterica* subsp. *enterica* serovars Dublin and Enteritidis, suggesting that to elaborate further on the hypothesis of RM systems being involved in the evolution of *Salmonella enterica* subsp. *enterica*, either a collection of closely related serovars or a more comprehensive data set with multiple representatives from each serovar could be assessed to expand on the hypothesis that the evolution of subgroups of *S. enterica* subsp. *enterica* RM systems could have stronger links between their genomic evolution and the presence of RM systems compared to the lack of association for the entire subspecies *enterica*.

## MATERIALS AND METHODS

### *Salmonella* strains.

From an in-house strain collection at the Technical University of Denmark, National Food Institute (DTU FOOD), a subcollection of 68 *S. enterica* subsp. *enterica* isolates with global origins and a focus on multidrug resistance was submitted to the 100K Food Pathogen Genome Project (http://100kgenome.vetmed.ucdavis.edu/, NCBI BioProject accession number PRJNA186441) for WGS. Subsequently, the genomes from that project were merged with a genomic collection consisting of 105 *Salmonella* strains mainly originating from the American Type Culture Collection, often pansusceptible, which were sequenced by the U.S. Food and Drug Administration Center for Food Safety and Applied Nutrition (FDA-CFSAN) and U.S. Department of Agriculture (USDA) (1), and with another 48 publically available *Salmonella* genomes retrieved from the European Nucleotide Archive for inclusion in this study. The final data set of 216 *S. enterica* subsp. *enterica* genomes was constructed with a focus on diversity and included a total of 97 different *Salmonella* serovars. Additionally, five genomes of four other subspecies were included in the data set to form an outgroup. This data set might have a built-in bias with respect to the selective criteria, e.g., susceptibility to antimicrobial agents. Full genomic information is shown in Table S1 in the supplemental material.

### Whole-genome sequencing.

Nutrient agar sticks containing the 68 in-house *Salmonella* isolates were dispatched to the School of Veterinary Medicine, UC Davis, CA, USA, in relation to the 100K Foodborne Pathogen Genome Project. The genomic DNAs (gDNAs) were extracted from overnight cultures using KAPA enzyme lysis buffer and the DNeasy blood and tissue kit from Qiagen. The gDNAs were subsequently fragmented to average sizes of 200 to 450 bp using the Diogenode Bioruptor NGS or Covaris E220. Sequencing libraries were prepared using the KAPA high-throughput (HTP) library preparation kit for Illumina platforms. Briefly, the fragmented gDNAs were adenylated, end paired, and ligated to NEXTflex-96 DNA bar code-indexed sequencing adaptors (Bioo Scientific). Following the ligation, indexed double-stranded DNAs (dsDNAs) were selected by size by using AmPure beads and then amplified by PCR. The indexed sequencing libraries were pooled and sequenced on an Illumina HiSeq 2000 with PE100 plus index read. The raw reads of the 68 sequenced genomes, received from UC Davis, were assembled using the Assembler 1.0 pipeline from the CGE, available on http://cge.cbs.dtu.dk/services/all.php, which is based on the Velvet algorithms for *de novo* short-read assembly. A complete list of genomic sequence data is available in Table S1 in the supplemental material.

### Construction of core and pangenome plot and pan- and core genome trees.

Open reading frames (ORFs) were predicted on the contigs by using the Prodigal software (46). The same gene predictor was subsequently used to eliminate biases in annotation quality and to standardize the genes found in all genomes (47).

The predicted genes were translated into amino acid sequences and aligned all-against-all using BLASTP (48). Two genes were determined as a gene pair if the alignment length was at least 50% of the longest sequence and more than 50% of the aligned sequences were reported as identical (the 50/50 rule). As a member of a gene pair can be a member of other gene pairs by this method, all gene pair-sharing members were subsequently combined into a gene family, which ensures that each gene will belong exclusively to only one gene family (28, 47, 49–53).

### Pan- and core genome plot.

The core and pangenome plot was constructed by comparing the gene families from all genomes. The pangenome was constructed from the union of the genes from the genomes under consideration, while the core genome was built from the intersection of gene families shared by every genome under analysis (27, 28).

### Pangenome tree.

The pangenome tree was reconstructed from a matrix consisting of gene families (rows) and genomes (columns). In the matrix, the absence and presence of genes across the genomes were represented by 0’s and 1’s, respectively. The genomes were clustered using hierarchical clustering of the relative Manhattan distance between genomes, and the bootstrap values were calculated to represent the confidence level of branches (27, 54).

### Core genome tree.

The core genes were aligned in a BLAST-like manner, using BLAT version 35 (55), to the predicted genes of each genome. The genes found in all genomes were then aligned using MUSCLE version 3.8.31 (56) and concatenated to a single alignment. Five hundred resamples of the alignment were created with Seqboot version 3.67 (part of the PHYLIP package [57]).

A gene was considered identified according to the 50/50 rule. DNADist (57) was employed to calculate the genomic distances from the initial alignment, as well as each of the 500 resamples. FastMe (58) was used to calculate trees from the distance matrices. The tree from the original alignment was compared to the 500 trees created from the resamples by using CompareToBootstrap (59). The final tree was visualized with FigTree (http://tree.bio.ed.ac.uk/software/figtree/).

### Construction of RM-Finder and SPI-Finder.

To be able to analyze the genomes for their content of RM systems and SPIs, two publically available online tools were developed. Both tools, Restriction-ModificationFinder (https://cge.cbs.dtu.dk/services/Restriction-ModificationFinder/) and SPI-Finder (https://cge.cbs.dtu.dk/services/SPIFinder/), were built on a BLAST-based methodology for detection of genes from customized databases, originally developed by Zankari et al. for *in silico* detection of acquired resistance genes (60). The tools were developed to process both preassembled genomes and data of raw reads from different sequencing platforms, with user selection parameters of minimum percent identity (%ID) and minimum length. The default settings were chosen as minimum %ID at 95%, to avoid noise and fragments of the genes, and a minimum length of 60%, to be able to detect genes in the start or end of contigs from bad assemblies.

The database behind RM-Finder originates from the authoritative source REBASE (20, 33) and includes type I to IV restriction genes, methyltransferases, and specificity units. The database is categorized into two groups, one only including genes with confirmed functions and one where putative genes are included. The RM-Finder database is updated monthly.

The *Salmonella enterica* records from the PAthogenicity Island DataBase (PAIDB) served as inspiration for the customized database behind SPI-Finder (61, 62).

Extensive explanations of the output can be accessed on separate tabs at the Web sites for the online tools, https://cge.cbs.dtu.dk/services/Restriction-ModificationFinder/ and https://cge.cbs.dtu.dk/services/SPIFinder/.

### Identification of RMS genes, plasmid replicons, SPIs, and antimicrobial resistance.

To analyze the content of RM systems in the 221 genomes, all ORFs were submitted to Restriction-ModificationFinder version 1.1, available on the CGE website (https://cge.cbs.dtu.dk/services/Restriction-ModificationFinder/), to identify restriction (R), methyltransferase (M), and specificity (S) genes. Subsequently, the R, M, and S genes identified were individually inspected to form RM systems, and putative systems were assigned when all genes required were present and adjacent on the contig, even if truncated or frame shifted. For systems with unknown specificity, systems were assigned according to the specificity subunit present. However, incomplete systems were investigated for truncated genes. Additionally, contigs with incomplete systems were inspected by BLAST against REBASE, and putative genes for completion were revealed. Based on the predicted recognition sequences, the systems were merged and named according to the type of system (see Table S2 in the supplemental material). An RM system presence/absence matrix was constructed in R version 2.14 (http://cran.r-project.org/bin/windows/base/old/2.14.0/) with hierarchical clustering and Euclidean distance (Fig. 1; see also Fig. S1).

A phylogeny of the type III RM systems was created by extracting the ORFs for the methyltransferases and the restriction enzymes from the genomes, and the tree was constructed as described for the core genome tree and visualized by FigTree (see Fig. S4 in the supplemental material).

The 221 draft genomes were analyzed for their content of plasmid replicons, pathogenicity islands, and antimicrobial resistance genes by using the CGE Web tools PlasmidFinder 1.1 (21), SPIFinder 1.0 (https://cge.cbs.dtu.dk/services/SPIFinder/), and ResFinder 2.0 (60) with %ID of 80.00 and minimum length at 60.00%. The content of plasmid replicons and pathogenicity islands was interpreted by Circos plots (http://circos.ca/). For the antimicrobial resistance genes, a presence/absence matrix was constructed as described for the RM systems (see Fig. S2 in the supplemental material).

### Data availability.

Data underlying the findings are shared on figshare (https://dx.doi.org/10.6084/m9.figshare.c.3247351) (63), as follows: gene clusters of the 16,375 *Salmonella* pangenes for all genomes (Pan-gene_clusters.tar.gz), gene clusters of the 2,138 *Salmonella* core genes for all genomes (Core-gene_clusters.tar.gz), concatenated alignment for the core genome tree (Concatenated_alignment_core-genome_tree.fa), raw tree file for the core genome tree (Core-genome_tree.newick), raw tree file for the pangenome tree (Pan-genome_tree.newick), output information from Restriction-ModificationFinder for each of the 221 genomes (RMFinder_output.xlsx), concatenated alignment of type III RM systems (Concatenated_alignment_typeIII_RMsystems.fa), and raw tree file for type III RM systems (Type_III_tree.newick). The 68 *Salmonella* genomes sequenced as part of the 100K Foodborne Pathogen Genome Project (http://100kgenome.vetmed.ucdavis.edu/) are available under NCBI BioProject accession number PRJNA186441; individual accession numbers are listed in Table S1.
