# Supplementary figures and images for: Is the Evolution of Salmonella enterica subsp. enterica Linked to Restriction-Modification Systems?
Source: mSystems. 2016 Jun 21;1(3):e00009-16. doi: 10.1128/mSystems.00009-16 (PMC5069764; doi:10.1128/mSystems.00009-16)

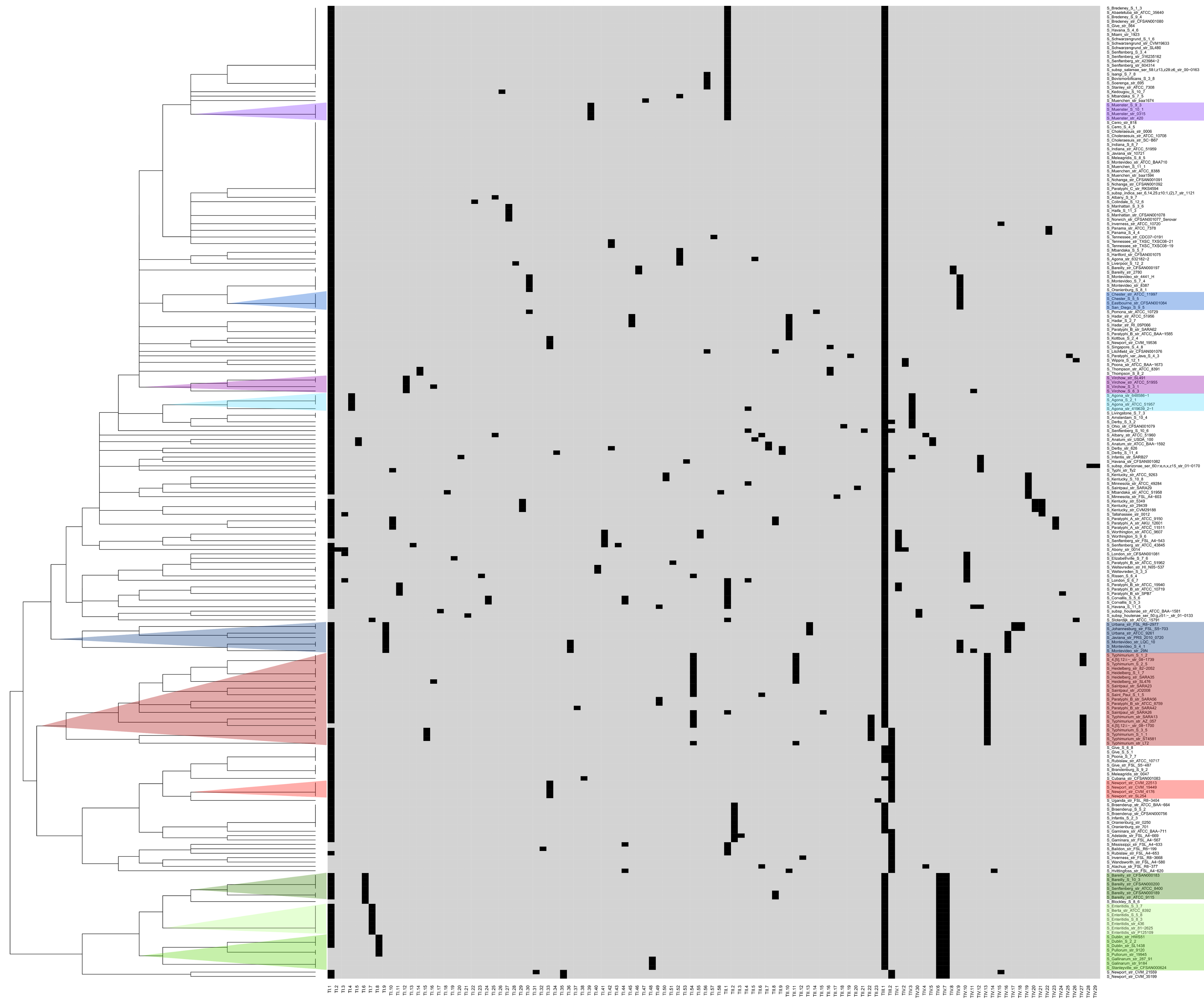

Supplement: Figure S1 [file sys003162032sf3.tif]

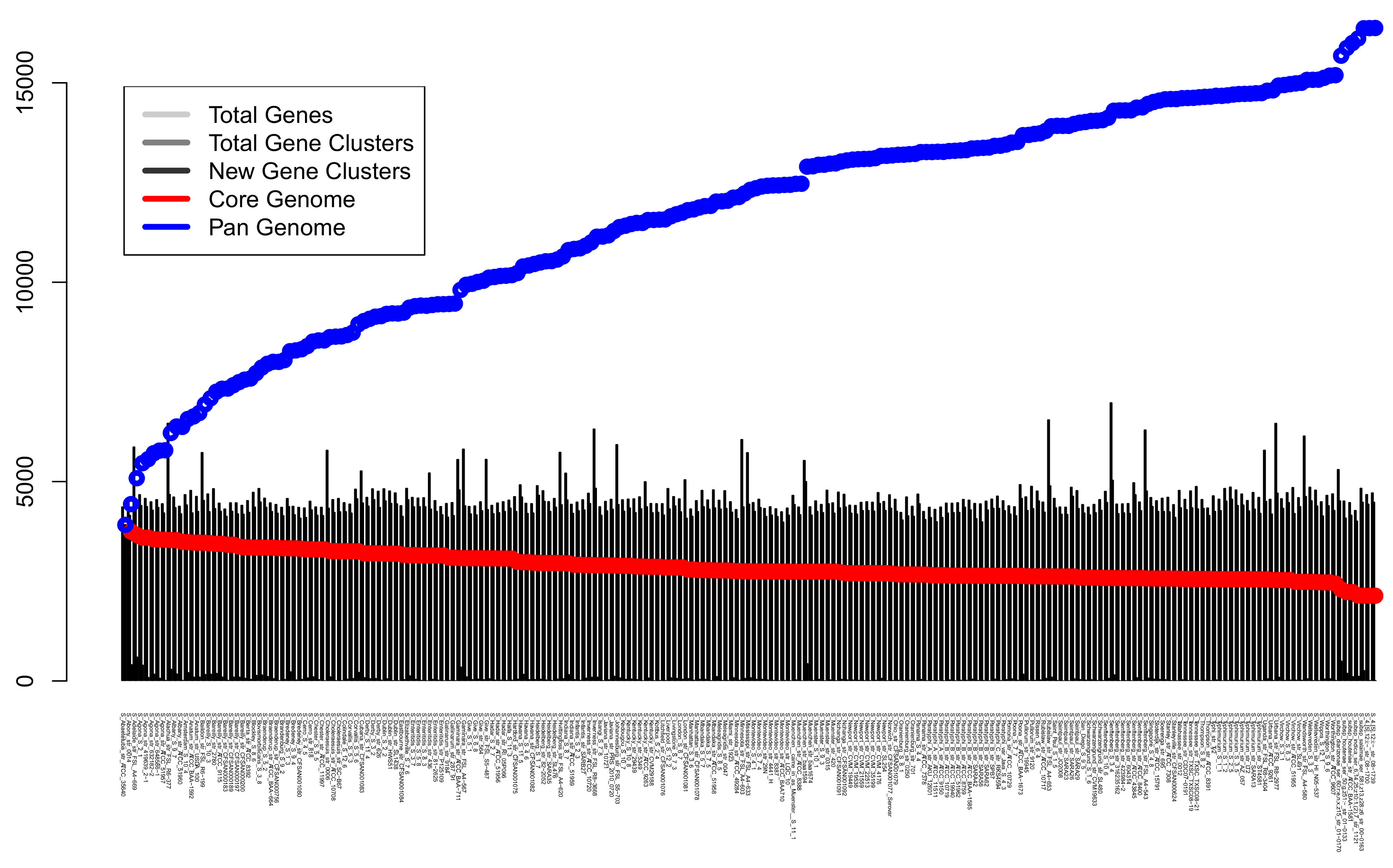

Supplement: Figure S2 [file sys003162032sf4.tif]

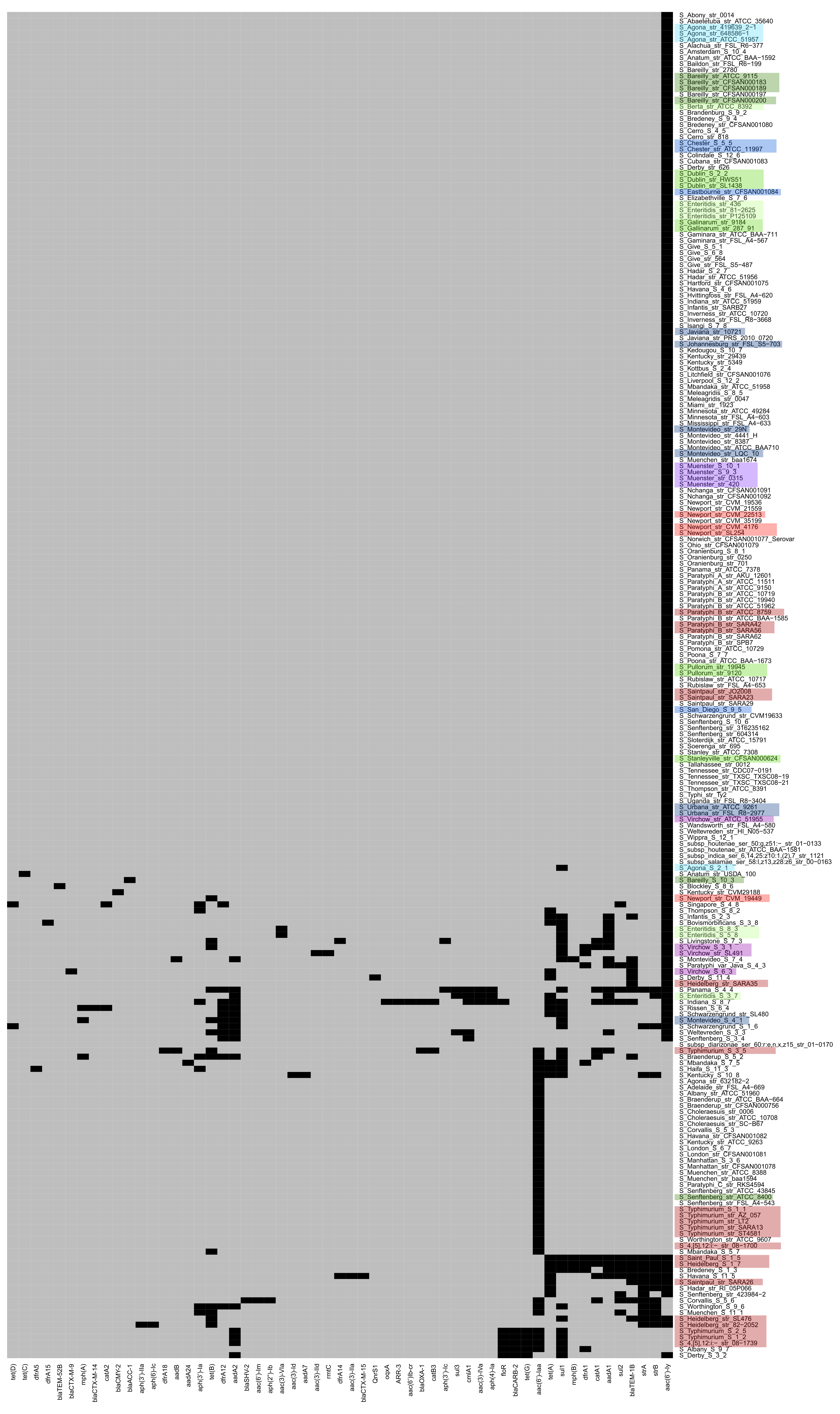

Supplement: Figure S3 [file sys003162032sf5.tif]

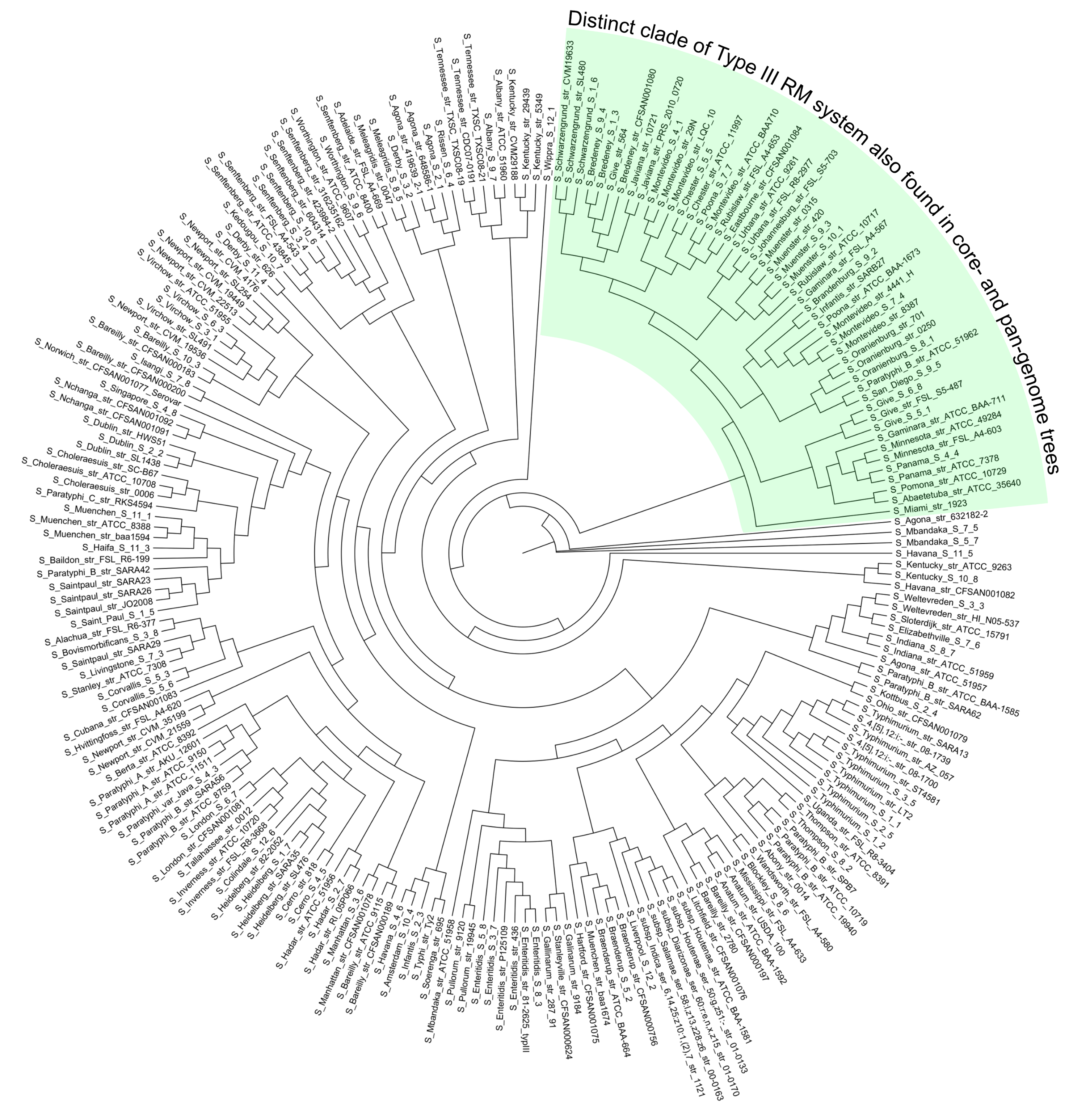

Supplement: Figure S4 [file sys003162032sf6.tif]
